# Size-Associated Partitioning of Oral Polystyrene Particles Between Intestinal Capillaries and Lacteals Determines Systemic and Lymph Node Distribution in Mice

**DOI:** 10.3390/nano16201278

**Published:** 2026-10-09

**Authors:** Huaiqiang Yi, Yangxia Zhang, Huijie Jia, Jiangning Chen, Zhen Huang

**Affiliations:** 1 State Key Laboratory of Pharmaceutical Biotechnology, School of Life Sciences, Nanjing University, Nanjing 210023, China; huaiqiang.yi@smail.nju.edu.cn (H.Y.); 602024300068@smail.nju.edu.cn (Y.Z.); 602023300021@smail.nju.edu.cn (H.J.); 2 State Key Laboratory of Analytical Chemistry for Life Sciences, Nanjing University, Nanjing 210023, China; 3 NJU Xishan Institute of Applied Biotechnology, Wuxi 214101, China

**Keywords:** polystyrene particles, intestinal capillaries, lacteals, lymphatic transport, mesenteric lymph nodes

## Abstract

Micro- and nanoplastics are widespread environmental contaminants; however, the intestinal routes governing their entry into systemic circulation remain unclear. Here, we used commercially available fluorescently labeled spherical polystyrene (PS) particles as a model system to investigate the intestinal transport of 100, 500 and 1000 nm particles in mice. We combined whole-mount imaging of intestinal villi with blood and organ kinetic analyses, mesenteric imaging and modulation of lacteal endothelial junctions. The whole-blood concentration of 100 nm particles peaked at 1 h, followed by peak liver and kidney fluorescence at 2 h. In contrast, 500 and 1000 nm particles preferentially localized to lacteals and mesenteric lymphatic vessels, accumulated more strongly in mesenteric lymph nodes and showed delayed blood and organ peaks at 6 h. Shifting lacteal junction organization from discontinuous button-like junctions toward continuous zipper-like junctions with the Rho-associated kinase inhibitor Y-27632 reduced the lymph node accumulation of the larger particles, supporting a functional role for lacteal permeability in their uptake and downstream transport. Repeated exposure to the corresponding unmodified PS particles was associated with lower body weight at day 30, as well as mesenteric lymph node enlargement and cellular disorganization. These lymph node alterations were accompanied by increased mRNA expression of the pro-inflammatory cytokine genes *Ifng*, *Il1b* and *Tnf*, consistent with a local inflammatory response. Together, these findings demonstrate size-associated partitioning of orally administered PS model particles between intestinal blood and lymphatic routes and provide a framework linking intestinal entry with subsequent systemic distribution, while identifying local mesenteric lymph node responses following repeated exposure.

## 1. Introduction

Plastics are widely used in industrial, agricultural and consumer applications, and their increasing production, extensive use and improper disposal have contributed to widespread environmental contamination by microplastics (MPs; 1 µm–5 mm) and nanoplastics (NPs; <1 µm) [1,2]. Micro- and nanoplastics may originate as primary particles intentionally manufactured at small sizes or as secondary particles generated through the fragmentation and weathering of larger plastic products [3]. Their widespread occurrence in food and drinking water makes ingestion an important route of human exposure [4,5]. Polystyrene (PS) warrants particular toxicological attention because it is widely used in food-contact materials, packaging and disposable products [6,7]. Experimental studies have associated oral exposure to PS particles with intestinal barrier disruption, oxidative stress, epithelial apoptosis, impaired mitophagy, gut microbiota dysbiosis and inflammatory responses [8,9,10]. These reported adverse effects underscore the need to clarify how ingested PS particles cross the intestinal barrier and subsequently distribute within the body.

Particle size is an important factor influencing the gastrointestinal fate of ingested plastic particles. Compared with micrometer-sized particles, NPs are more strongly influenced by Brownian motion and have higher surface-area-to-volume ratios, which may promote their interactions with biological components and facilitate cellular uptake and barrier translocation [11,12]. Studies using PS model particles have suggested that gastrointestinal absorption generally decreases with increasing particle size, while more recent intestinal epithelial models have demonstrated size-associated differences in cellular internalization and transepithelial transport [13,14]. However, this relationship is also influenced by surface chemistry, aggregation state, protein-corona formation and the cellular composition of the experimental model [15,16]. Moreover, previous studies have primarily examined epithelial translocation or subsequent accumulation in systemic organs [17]. Direct in vivo evidence showing whether particles of different sizes preferentially partition into distinct transport compartments after crossing the intestinal epithelium remains limited.

Each intestinal villus contains two anatomically adjacent transport systems: blood capillaries and a central lacteal [18,19]. These vessels normally transport water-soluble and lipid-associated materials, respectively, suggesting that particles entering either compartment may follow different downstream routes [20,21,22]. Entry into villous blood capillaries may provide rapid access to the portal and systemic circulation, whereas lacteal uptake may direct particles through mesenteric lymphatic vessels, delay their systemic appearance and increase their accumulation in mesenteric lymph nodes [23,24,25]. Despite these distinct transport consequences, how PS particles of different sizes partition between villous blood capillaries and lacteals remains poorly understood [26]. Lacteal endothelial junctions form a dynamically regulated permeability barrier characterized by discontinuous button-like and continuous zipper-like junctions. Whether this junctional organization regulates the intestinal uptake and downstream lymphatic transport of larger PS particles has not yet been established [27,28].

Although biomedical nanocarrier studies frequently emphasize particles smaller than 100 nm to optimize cellular uptake and systemic circulation, orally encountered nanoplastics encompass a broader size range extending toward the micrometer scale [29,30]. We therefore used commercially available fluorescently labeled spherical PS particles of 100, 500 and 1000 nm as a standardized model system. These sizes represent a nanoscale particle, an intermediate submicrometer particle and a particle at the operational nano–microplastic boundary, respectively. We hypothesized that PS particles of different sizes would exhibit preferential intestinal transport routes, with smaller particles localizing predominantly to villous blood capillaries and appearing more rapidly in the systemic circulation, whereas larger particles would preferentially enter lacteals, resulting in delayed systemic distribution and greater mesenteric lymph node accumulation. We further hypothesized that reducing lacteal permeability by shifting endothelial junctions toward a zipper-like organization would decrease the lymphatic uptake of larger particles. To test these hypotheses, we combined whole-mount imaging of intestinal villi with blood and organ kinetic analyses, mesenteric and lymph node imaging, and pharmacological modulation of lacteal endothelial junctions. We also evaluated whether repeated oral exposure was associated with morphological and inflammatory changes in mesenteric lymph nodes.

## 2. Materials and Methods

### 2.1. Animals

Male C57BL/6JNifdc mice (6–8 weeks old) were obtained from Beijing Vital River Laboratory Animal Technology Co., Ltd. (Beijing, China). The mice were housed in a specific pathogen-free facility at 22–24 °C under a 12 h light/12 h dark cycle, with ad libitum access to standard laboratory chow and drinking water [31].

### 2.2. PS Particle Preparation and Characterization

Orange fluorescent polystyrene particles with nominal diameters of 100 nm (Cat. No. 7-2-0010), 500 nm (Cat. No. 7-2-0050), and 1000 nm (Cat. No. 7-2-0100) and the corresponding unmodified particles (Cat. Nos. 6-1-0010, 6-1-0050, and 6-1-0100, respectively) were purchased from Tianjin BaseLine ChromTech Research Center (Tianjin, China). Fluorescent particles were used for the biodistribution and uptake experiments, whereas unmodified particles were used for the repeated-exposure study [32]. Particle morphology was characterized using scanning electron microscopy (SEM; Regulus 8100, Hitachi High-Technologies Corporation, Tokyo, Japan) and transmission electron microscopy (TEM; JEM-2100F, JEOL Ltd., Tokyo, Japan). SEM and TEM imaging were performed by Wuhan Servicebio Technology Co., Ltd. (Wuhan, China) and Shanghai Baitoubaizhong Testing Technology Co., Ltd. (Shanghai, China), respectively. PS particles were diluted to a final concentration of 100 μg/mL in pure water or pure water containing 10% (*v*/*v*) FBS (Cat. No. F103-01; Vazyme Biotech, Nanjing, China) and incubated at 37 °C for 30 min. Their hydrodynamic diameter, polydispersity index (PDI) and zeta potential were subsequently measured using a 90Plus PALS particle size and zeta potential analyzer (Brookhaven Instruments Corporation, Holtsville, NY, USA).

### 2.3. Single-Dose Oral Administration for Transport and Biodistribution Analysis

Mice were fasted for 8 h before oral gavage of PS particles. The selected dose was based on previously published oral PS-particle studies using comparable dosing regimens to investigate particle uptake, biodistribution and biological effects [17,33,34]. For the transport and biodistribution experiments, mice were randomly assigned to the control group or one of three particle-treatment groups. Mice in the particle-treatment groups received a single oral dose of fluorescent PS particles (100, 500 or 1000 nm; 15 mg/kg) freshly resuspended in 200 μL of PBS, whereas control mice received 200 μL of PBS alone. For blood kinetic analysis, 40 μL of tail-vein blood was collected into anticoagulant-treated tubes before gavage (0 h) and at 1, 2, 4, 6, 8 and 12 h after administration.

For ex vivo fluorescence imaging, the mice were euthanized at the indicated time points after gavage, and the small intestine, heart, liver, spleen, lungs, kidneys, mesentery and mesenteric lymph nodes were collected. Jejunal tissue and mesentery were collected at 2 h for whole-mount immunofluorescence, mesenteric lymph nodes were collected at 4 h for whole-mount immunofluorescence, and the heart, liver, spleen, lungs, and kidneys were collected at 6 h for cryosection imaging. For the lacteal junction inhibition experiment, Y-27632 (Cat. No. 688000, Sigma-Aldrich, St. Louis, MO, USA; 20 μg/g) was dissolved in PBS and administered intraperitoneally 2 h before gavage to promote lacteal junction zippering [20]. Control mice received an equal volume of PBS via the same route. The small intestine and mesenteric lymph nodes were collected at 2 and 4 h after PS-particle administration, respectively.

### 2.4. Repeated Oral Exposure and Biological Response Assessment

To evaluate the biological effects of repeated exposure, mice were randomly assigned to the PBS-treated control group or one of three particle-treatment groups. Mice in the particle-treatment groups received unmodified PS particles (100, 500 or 1000 nm; 15 mg/kg body weight/day) suspended in 200 μL of PBS by oral gavage once daily for 30 consecutive days, whereas control mice received 200 μL of PBS following the same administration schedule. The exposure dose and duration were selected based on previously published studies [17,33,35]. Body weight was recorded every 5 days. At the end of the exposure period, mesenteric lymph nodes were photographed, the maximum diameter of the largest node from each mouse was measured using Fiji (ImageJ, version 2.14.0, NIH, Bethesda, MD, USA), and the lymph nodes were collected for histological and qRT-PCR analyses.

### 2.5. Whole-Blood Fluorescence Quantification

To quantify circulating particles across all particle sizes using a single procedure, 10 μL of anticoagulated whole blood was diluted with 90 μL of PBS in a black 96-well plate. Fluorescence was measured using a Spark multimode microplate reader (Tecan Trading AG, Männedorf, Switzerland) at excitation and emission wavelengths of 510 and 610 nm, respectively. Blank-subtracted fluorescence values were converted to whole-blood particle concentrations using separate, size-specific matrix-matched calibration curves prepared by spiking known concentrations of each particle formulation into untreated mouse blood and processing the standards identically to the corresponding study samples [36].

### 2.6. Ex Vivo Fluorescence Imaging

Freshly excised tissues collected at indicated time points after gavage were imaged using an IVIS Spectrum system (PerkinElmer, Waltham, MA, USA) at excitation and emission wavelengths of 535 and 580 nm, respectively. Tissues from PBS-treated mice were used to determine background fluorescence under identical acquisition conditions [37].

### 2.7. Whole-Mount Immunofluorescence Staining of the Jejunum and Mesentery

Jejunal segments were fixed in 4% formaldehyde for 6 h and subsequently dehydrated in 100% methanol for 20 min. Freshly isolated mesentery was maintained in its native anatomical orientation, fixed in 4% paraformaldehyde for 6 h, and dehydrated in 100% methanol for 20 min. For whole-mount staining of lacteal junctions, jejunal tissues were fixed in 1.6% formaldehyde at room temperature for 43 min, followed by incubation in 100% methanol on ice for 20 min. Jejunal samples were then blocked for 1 h at room temperature in PBS containing 5% normal donkey serum, 0.15% Triton X-100, and 3% BSA [38]. Mesenteric samples were blocked for 8 h in PBS supplemented with 5% normal goat serum, 0.15% Triton X-100, and 3% BSA [39].

Both tissue samples were sequentially incubated with the specified primary antibodies at 4 °C overnight, followed by the corresponding secondary antibodies at room temperature for 45 min. After the immunolabeling procedure, all samples were post-fixed in 4% (*w*/*v*) formaldehyde for 10 min to stabilize the antigen–antibody complexes. After fixation, the intestinal wall was dissected under a stereomicroscope into thin sheets containing a single layer of villi. Jejunal and mesenteric samples were mounted in antifade mounting medium (Cat. No. P0131; Beyotime Biotechnology, Shanghai, China) and imaged using an LSM 980 confocal laser-scanning microscope (ZEISS, Oberkochen, Germany). The antibodies used are listed in Table A1.

### 2.8. Cryosection Immunofluorescence Staining

Harvested tissues were fixed in 4% formaldehyde for 5–10 min, embedded in O.C.T. compound (Cat. No. 4583; Sakura Finetek, Torrance, CA, USA), and cut into 5 μm sections using a CM1900 cryostat (Leica Biosystems Nussloch GmbH, Nussloch, Germany). After fixation and blocking, the sections were incubated with primary antibodies overnight at 4 °C and with secondary antibodies for 45 min at room temperature. After each antibody incubation step, the sections were washed three times with PBS for 5 min, mounted in DAPI-containing antifade mounting medium (Cat. No. P0131; Beyotime Biotechnology), and imaged using an LSM 980 confocal microscope (Carl Zeiss Microscopy GmbH, Jena, Germany) [40]. The antibodies used are listed in Appendix A Table A1.

### 2.9. Lacteal Morphology Analysis

Lacteal morphology was quantified using Fiji (ImageJ, version 2.14.0, NIH, Bethesda, MD, USA) [41]. VE-cadherin-positive junctions within LYVE-1-positive lacteals were classified as button-like or zipper-like using established criteria with minor modifications. Button-like junctions were defined as discontinuous contacts oriented nonparallel to the cell–cell border and associated with the characteristic oak-leaf morphology of lymphatic endothelial cells. Zipper-like junctions were defined as continuous, linear contacts aligned parallel to the cell–cell border [42]. The lengths of the button-like and zipper-like junctions were measured and expressed relative to the total junction length. Lacteal length was measured from the villus base to the lacteal tip. Ten lacteals were analyzed per mouse. Measurements from individual lacteals were averaged for each mouse, and the resulting per-animal mean was used as the biological replicate for statistical analysis.

### 2.10. Hematoxylin and Eosin Staining

Hematoxylin and eosin staining was performed by AiFang Biotechnology Co., Ltd. (Changsha, China). Mesenteric lymph nodes were fixed in 4% formaldehyde for 8 h, dehydrated through a graded ethanol series, embedded in paraffin, and sectioned at 5 μm. Sections were stained with hematoxylin and eosin (Leagene Biotechnology, Beijing, China) according to the manufacturer’s instructions and imaged using a KFBIO KF-FL-020 digital pathology scanner (Ningbo Konfoong Bioinformation Technology Co., Ltd., Ningbo, China) [43].

### 2.11. qRT-PCR Analysis

Total RNA was extracted from mesenteric lymph nodes collected after repeated PS-particle exposure using Invitrogen™ TRIzol™ Reagent (Cat. No. 15596018, Thermo Fisher Scientific, Carlsbad, CA, USA). RNA was reverse-transcribed into cDNA using the HiScript III RT SuperMix for qPCR (+gDNA wiper) (Cat. No. R323-01; Vazyme Biotech, Nanjing, China) according to the manufacturer’s instructions. Quantitative real-time PCR was subsequently performed using ChamQ Universal SYBR qPCR Master Mix (Cat. No. Q331-02, Vazyme Biotech, Nanjing, China) on a StepOne™ Real-Time PCR System (Thermo Fisher Scientific, Waltham, MA, USA). Relative mRNA expression levels of the inflammatory cytokine genes *Tnf*, *Ifng*, *Il6* and *Il1b* were calculated using the 2^−ΔΔCt^ method, with *Actb* as the reference gene [44]. The primer sequences are listed in Table A2.

### 2.12. Statistical Analysis

Statistical analyses were performed using GraphPad Prism (GraphPad Software, Version 10.1.2; Boston, MA, USA). Data normality was assessed using the Shapiro–Wilk test, and homogeneity of variance was evaluated using the Brown–Forsythe test before parametric group comparisons. For comparisons between two groups, normally distributed data with homogeneous variances were analyzed using a two-tailed unpaired Student’s *t*-test. When variances were unequal, Welch’s unpaired *t*-test was used. Non-normally distributed data were analyzed using the Mann–Whitney *U* test. For comparisons among three or more groups, normally distributed data with homogeneous variances were analyzed using ordinary one-way ANOVA followed by Tukey’s multiple-comparison test. When variances were unequal, Brown–Forsythe and Welch ANOVA followed by Dunnett’s T3 multiple-comparison test were used. Body weight trajectories were analyzed using repeated-measures two-way ANOVA followed by Sidak’s multiple-comparison test, as specified in the figure legends. Nonparametric comparisons among three or more groups were performed using the Kruskal–Wallis test followed by Dunn’s multiple-comparison test. Data are presented as the mean ± SEM, and sample sizes and statistical tests are specified in the corresponding figure legends.

## 3. Results

### 3.1. Physicochemical Characterization of PS Particles

**Figure 1:**
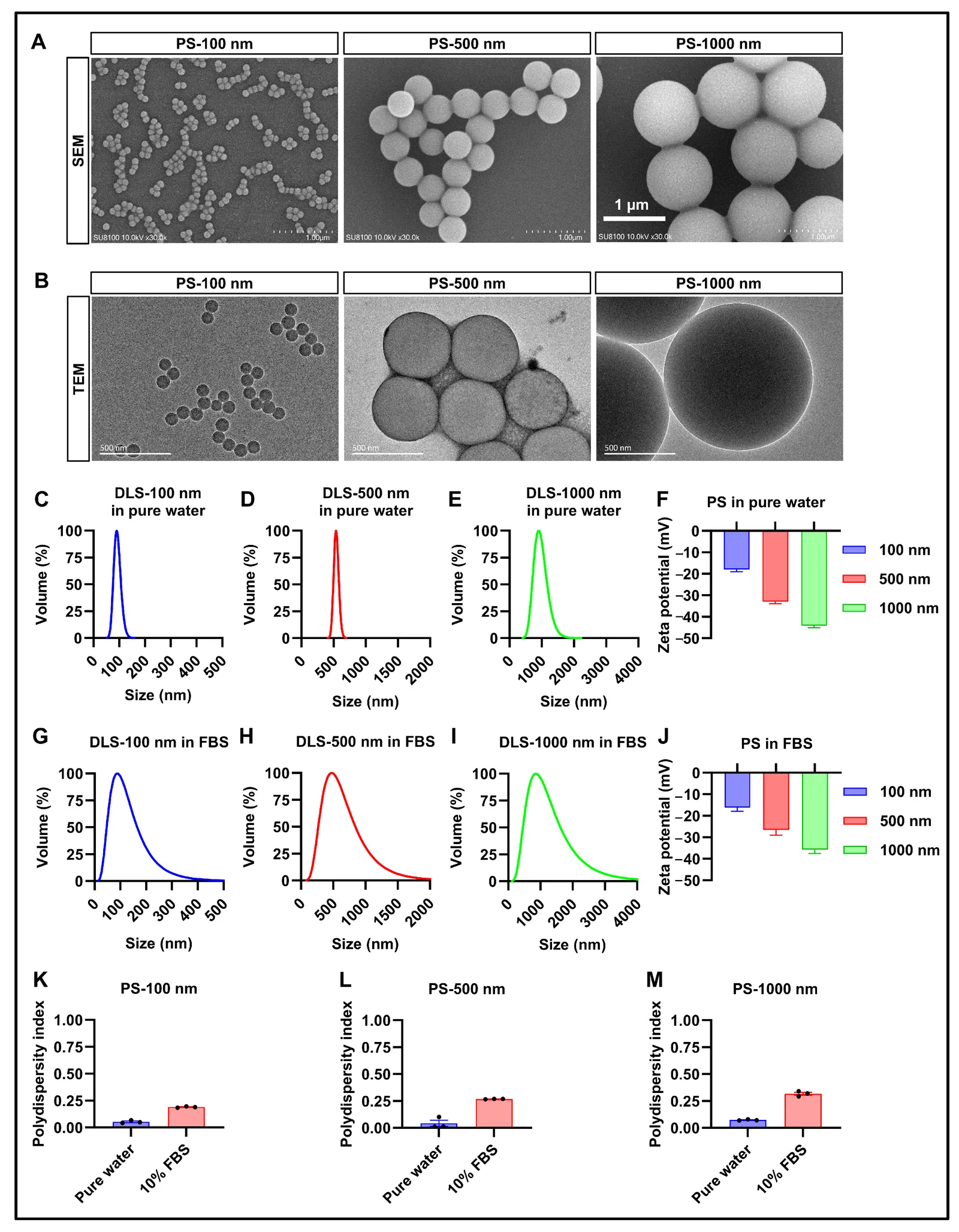
Physicochemical characterization of polystyrene particles of different sizes: (**A**) Representative scanning electron microscopy (SEM) images of polystyrene (PS) particles with nominal diameters of 100, 500 and 1000 nm; scale bars, 1 μm. (**B**) Representative transmission electron microscopy (TEM) images of the corresponding particles; scale bars, 500 nm. (**C**–**E**) Hydrodynamic-size distributions of the 100 nm (**C**), 500 nm (**D**) and 1000 nm (**E**) particles dispersed in pure water, as measured by dynamic light scattering (DLS). (**F**) Zeta potentials of the particles dispersed in pure water. (**G**–**I**) Hydrodynamic-size distributions of the 100 nm (**G**), 500 nm (**H**) and 1000 nm (**I**) particles after incubation in pure water containing 10% (*v*/*v*) fetal bovine serum (FBS) at 37 °C for 30 min. (**J**) Zeta potentials of the particles after incubation in the FBS-containing medium. (**K**–**M**) Polydispersity indices (PDIs) of the 100 nm (**K**), 500 nm (**L**) and 1000 nm (**M**) particles in pure water and the 10% FBS-containing medium. Each scattered dot on the bar graph represents one individual sample. The data are presented as the mean ± SEM from three independent experiments.

Scanning electron microscopy and transmission electron microscopy showed that the 100, 500 and 1000 nm PS particles were predominantly spherical, with smooth surfaces and well-defined boundaries (Figure 1A,B). Dynamic light scattering measurements in pure water yielded mean hydrodynamic diameters of 101.53 ± 1.20, 556.45 ± 4.57 and 1186.32 ± 9.03 nm, respectively, which were generally consistent with their nominal sizes (Figure 1C–E). The corresponding zeta potentials were −17.89 ± 1.15, −33.00 ± 0.93 and −44.13 ± 0.97 mV, respectively, with increasingly negative values observed for the larger particles (Figure 1F). After incubation in pure water containing 10% (*v*/*v*) FBS, all three particle formulations retained a single dominant hydrodynamic-size distribution peak, although the distributions shifted toward larger sizes and became broader than those measured in pure water (Figure 1G–I). The magnitude of the negative zeta potential decreased in the FBS-containing medium (Figure 1J), while the PDI increased for all three particle sizes (Figure 1K–M). No distinct secondary peak corresponding to a large aggregate population was observed during the examined incubation period.

### 3.2. Size-Associated Differences in Blood Appearance and Organ Distribution Kinetics

**Figure 2:**
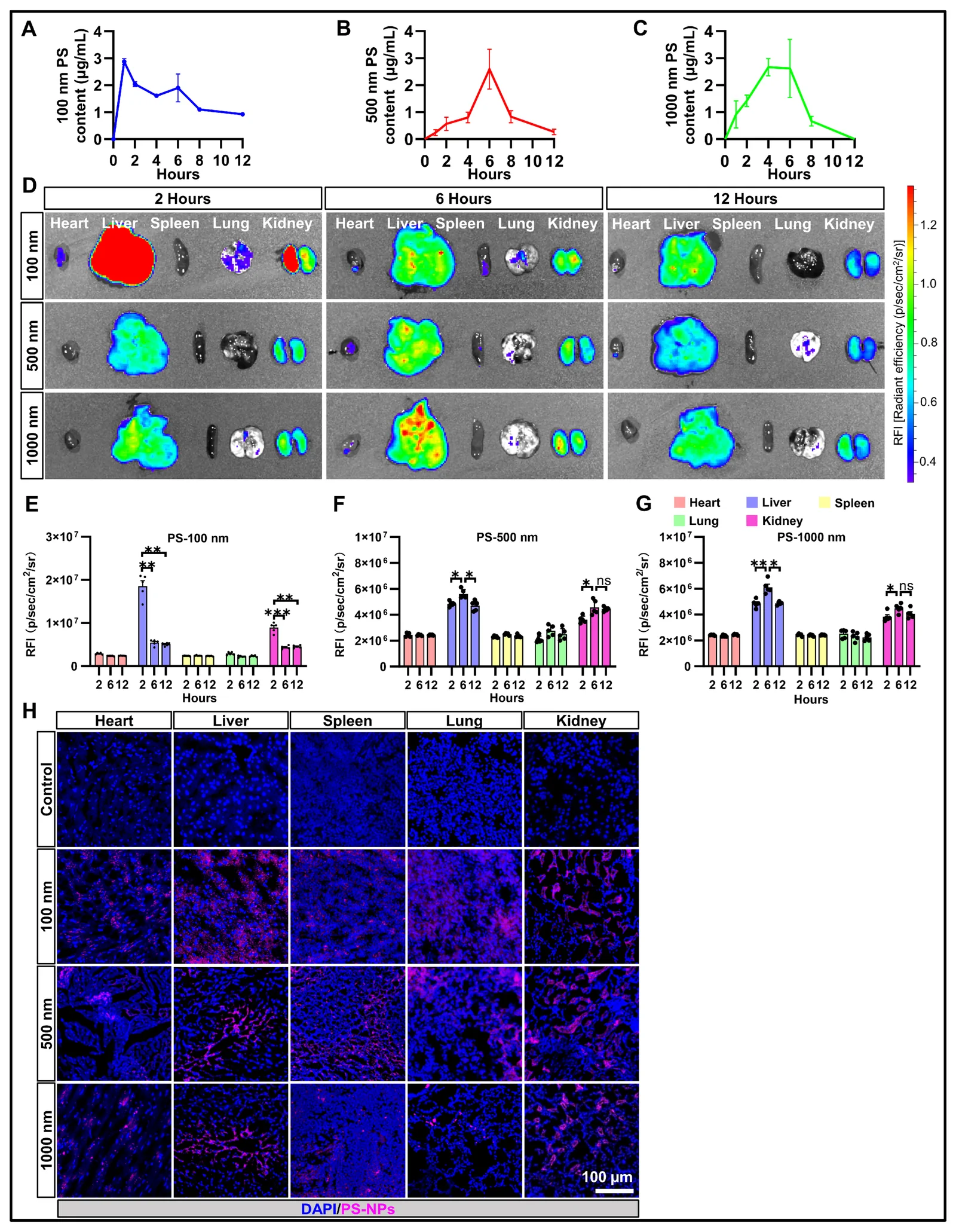
Blood appearance kinetics and tissue distribution of fluorescently labeled PS particles of different sizes: (**A**–**C**) Time-dependent blood concentrations of fluorescently labeled 100 nm (**A**), 500 nm (**B**), and 1000 nm (**C**) PS particles after oral gavage at 15 mg/kg (*n* = 10, 8, and 6 mice for the 100, 500, and 1000 nm groups, respectively). (**D**) Representative ex vivo fluorescence images of the heart, liver, spleen, lungs, and kidneys collected at 2, 6, and 12 h after oral gavage at the same dose. (**E**–**G**) Quantification of the fluorescence signals shown in (**D**) (*n* = 5 mice per group), each scattered dot on the bar graph represents one individual mouse. Color-coded bars denote quantitative in vivo fluorescence signals of 100, 500, and 1000 nm PS particles in different mouse organs, with one-to-one group mapping: heart (pink), liver (blue), spleen (yellow), lung (green), and kidney (magenta). (**H**) Representative fluorescence images showing the distribution of PS particles in the heart, liver, spleen, lungs, and kidneys at 6 h after oral gavage at 15 mg/kg (*n* = 3 mice per group; representative images are shown). DAPI (blue) and PS-NPs (purple); scale bar, 100 µm. The data are presented as the mean ± SEM. In panels (**E**–**G**), RFI values at 2, 6 and 12 h were analyzed separately for each organ within each particle-size group using Brown–Forsythe and Welch ANOVA followed by Dunnett’s T3 multiple-comparison test. All pairwise comparisons among the three time points were performed, with no comparisons made across organs or particle-size groups. For clarity, statistical annotations are shown only for the liver and lungs and display comparisons between the time point with the highest mean RFI and the other two time points. ns, not significant; * *p* < 0.05, ** *p* < 0.01 and *** *p* < 0.001.

Following oral gavage, fluorescence-derived blood concentrations increased transiently in all particle-size groups before decreasing (Figure 2A–C). The concentration of the 100 nm particles peaked at 1 h (approximately 2.88 μg/mL), whereas that of the 500 and 1000 nm particles peaked at 6 h (approximately 2.59 and 2.62 μg/mL, respectively). Although the measured peak concentrations were comparable, their timing differed markedly: the 100 nm particles appeared in the circulation more rapidly, whereas the larger particles showed delayed systemic entry.

Ex vivo organ imaging revealed a corresponding size-associated difference in distribution kinetics (Figure 2D–G). Particle-associated fluorescence accumulated predominantly in the liver and kidneys. The signals from the 100 nm particles in these organs peaked at 2 h, whereas those from the 500 and 1000 nm particles peaked at 6 h, mirroring their earlier and delayed appearance in blood, respectively. Organ fluorescence decreased by 12 h. At 6 h, particle-associated fluorescence was also observed in sections of the heart, liver, spleen, lungs and kidneys, indicating the presence of particle-associated fluorescence across major organs (Figure 2H).

### 3.3. The Small Intestine Was a Major Site of PS-Particle Uptake

**Figure 3:**
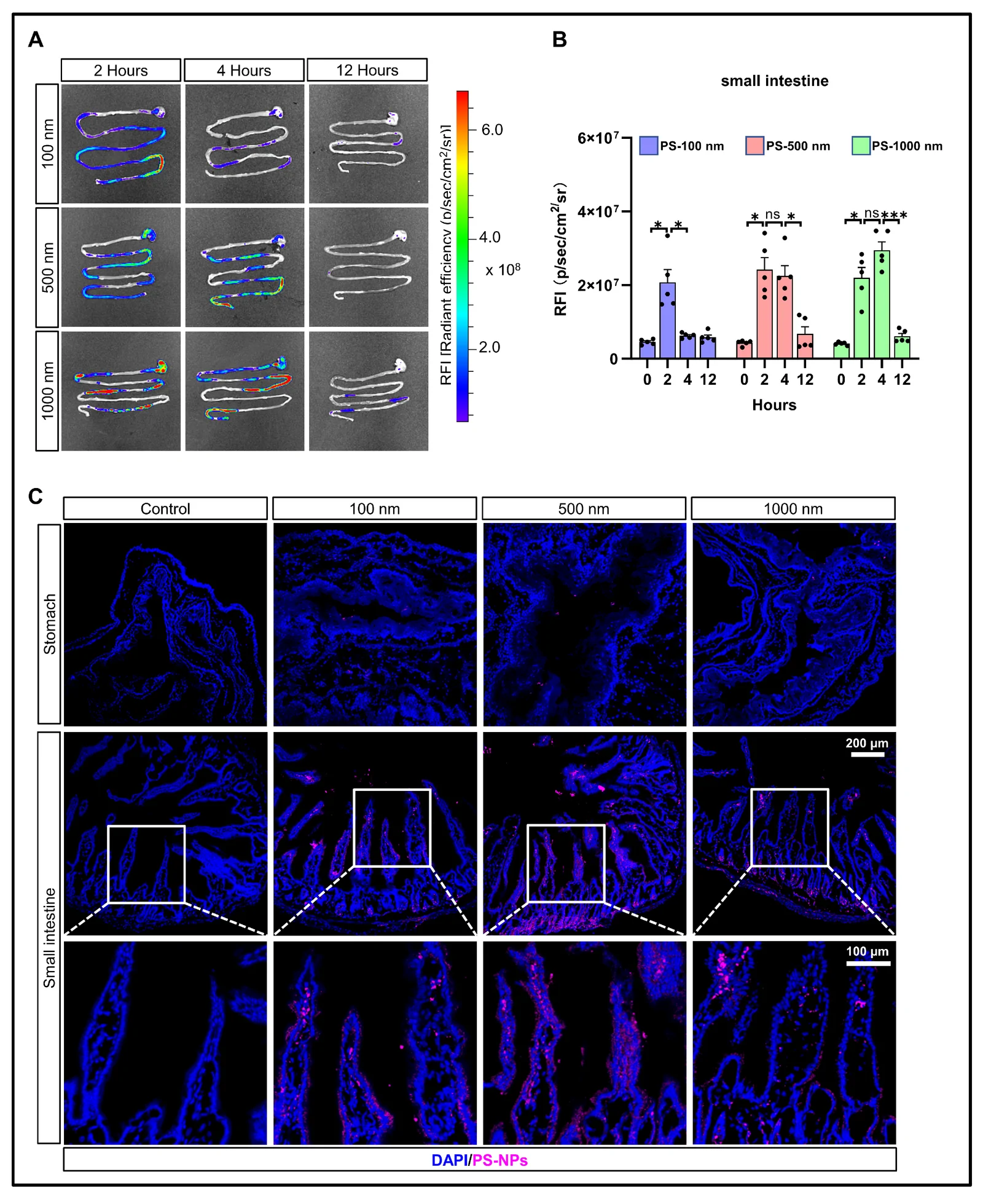
Gastrointestinal distribution of PS particles after oral gavage: (**A**) Representative ex vivo fluorescence images of the small intestine acquired at 2, 4, and 12 h after oral gavage of fluorescently labeled PS particles at 15 mg/kg. (**B**) Quantification of small-intestinal fluorescence at 0, 2, 4 and 12 h after oral gavage (*n* = 5 mice per group), each scattered dot on the bar graph represents one individual mouse. (**C**) Representative fluorescence images showing PS particles in the stomach and intestinal villi at 2 h after oral gavage at 15 mg/kg. DAPI (blue) and PS-NPs (purple); scale bars, 200 and 100 µm. The data are presented as the mean ± SEM. In panel (**B**), RFI values at 0, 2, 4 and 12 h were compared within each particle-size group using Brown–Forsythe and Welch ANOVA followed by Dunnett’s T3 multiple-comparison test. All pairwise comparisons among the four time points were performed, with no comparisons made across particle-size groups. ns, not significant; * *p* < 0.05 and *** *p* < 0.001.

Fluorescence imaging of gastrointestinal tissues revealed substantial particle retention in the intestine (Figure 3A,B). The intestinal signal from 100 nm particles reached its maximum at 2 h, whereas the 500 and 1000 nm particle signals peaked at 4 h. This temporal pattern was consistent with the earlier systemic appearance of the smaller particles and the delayed blood and organ peaks of the larger particles. The intestinal fluorescence in the 1000 nm group was greatest during the observation period, followed by a decrease at 12 h. The tissue sections collected 2 h after gavage contained little particle-associated fluorescence in the stomach but an abundant signal in the small intestine (Figure 3C). Together with their concurrent detection in blood and systemic organs, the predominant intestinal localization was consistent with the small intestine serving as an important site of particle uptake.

### 3.4. Size-Associated Preferential Intestinal Transport Routes of PS Particles

**Figure 4:**
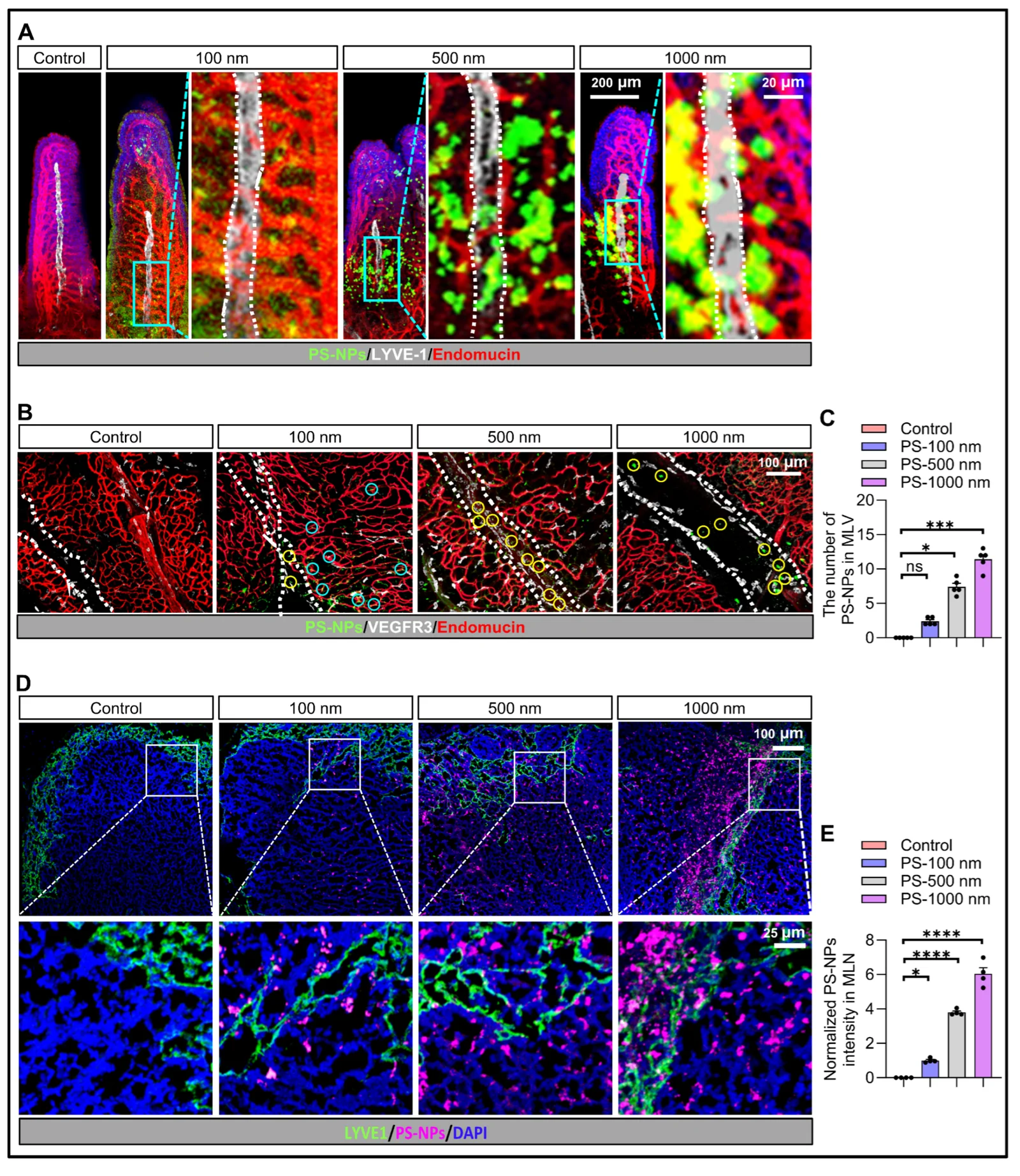
Whole-mount fluorescence imaging revealed the size-associated localization of PS particles in intestinal blood vessels and lacteals: (**A**) Representative whole-mount fluorescence images of intestinal villi collected at 2 h after oral gavage of PS particles (15 mg/kg). White dashed line demarcate the lacteal coverage, to clearly visualize PS nanoparticles entering the lacteals. PS-NPs (green), LYVE-1 (white) and Endomucin (red); scale bars, 200 and 20 µm. (**B**) Representative whole-mount fluorescence images of mesenteric blood and lymphatic vessels collected at 2 h after oral gavage. The dashed lines in the figure denote the coverage region of mesenteric lymphatic vessels. Blue circles indicate PS particles within mesenteric blood vessels, and yellow circles mark those in mesenteric lymphatic vessels. PS-NPs (green), VEGFR3 (white) and Endomucin (red); scale bar, 100 µm. (**C**) Quantification of fluorescent PS-particle counts in the mesenteric lymphatic vessels (MLV) shown in (**B**) (*n* = 5 mice per group), each scattered dot on the bar graph represents one individual mouse. (**D**) Representative whole-mount fluorescence images of mesenteric lymph nodes collected at 4 h after oral gavage. DAPI (blue), LYVE-1 (green) and PS-NPs (purple); scale bars, 100 and 25 µm. (**E**) Quantification of the PS-particle fluorescence intensity shown in (**D**), normalized to the mean value of the 100 nm group (*n* = 4 mice per group), each scattered dot on the bar graph represents one individual mouse. The data are presented as the mean ± SEM. Statistical significance was assessed using the Kruskal–Wallis test followed by Dunn’s multiple-comparison test in (**C**) and one-way ANOVA followed by Tukey’s multiple-comparison test in (**E**). ns, not significant; * *p* < 0.05, *** *p* < 0.001 and **** *p* < 0.0001.

Whole-mount imaging of jejunal villi showed that the 100 nm particles were observed mainly in association with endomucin-positive blood vessels, whereas the 500 and 1000 nm particles were observed mainly in association with LYVE-1-positive lacteals (Figure 4A). A similar size-associated distribution pattern was observed in the downstream mesentery, where 100 nm particles were detected mainly along blood vessels, while the larger particles were concentrated in lymphatic vessels (Figure 4B,C). Particle-associated fluorescence was also detected in mesenteric lymph nodes, with stronger signals observed in the 500 and 1000 nm groups (Figure 4D,E). Together, these observations show an association between particle size and preferential intestinal transport route, with 100 nm particles primarily associated with the vascular pathway and larger particles predominantly associated with the lacteal–mesenteric lymphatic–lymph node pathway.

### 3.5. Y-27632-Induced Lacteal Junction Zippering Reduced the Lymphatic Transport of Larger PS Particles

**Figure 5:**
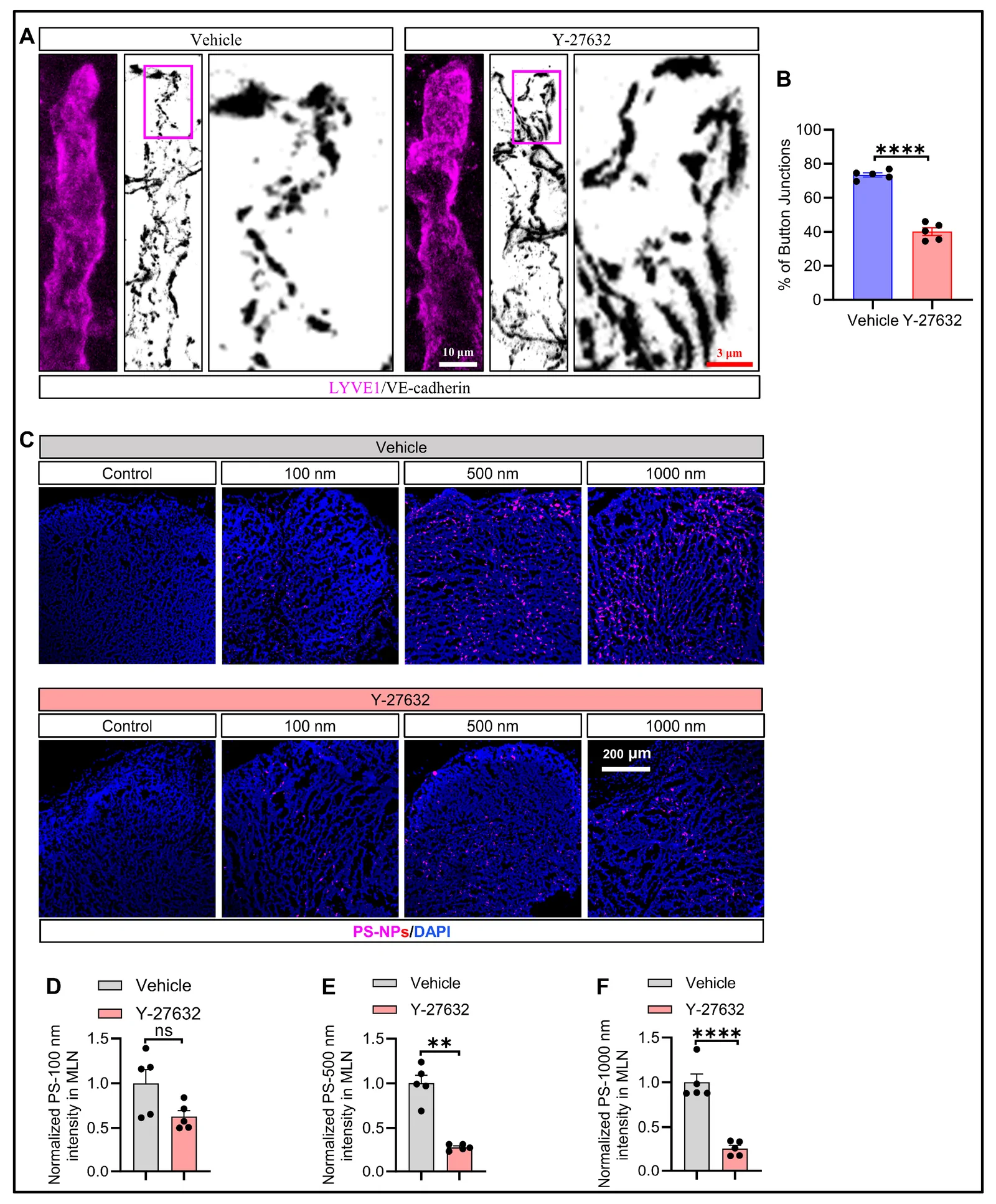
Y-27632-induced lacteal junction zippering reduces the mesenteric lymph node accumulation of 500 and 1000 nm PS particles: (**A**) Representative images of LYVE-1 and VE-cadherin staining in lacteals collected at 4 h after intraperitoneal administration of Y-27632 (20 µg/g). LYVE-1 (purple) and VE-cadherin (gray); scale bars, 10 and 3 µm. (**B**) Quantification of the proportion of button-like junctions relative to the total lacteal junction length shown in (**A**). Each symbol represents the mean value of ten randomly selected lacteals from an individual mouse; *n* = 5 mice per group. (**C**) Representative fluorescence images showing PS particle accumulation in mesenteric lymph nodes after Y-27632 treatment. Mice received Y-27632 (20 µg/g, intraperitoneally), followed by oral gavage of PS particles (15 mg/kg) 2 h later. Mesenteric lymph nodes were collected 4 h after gavage. DAPI (blue) and PS-NPs (purple); scale bar, 200 µm. (**D**–**F**) Quantification of the relative fluorescence intensity shown in (**C**) for 100 nm (**D**), 500 nm (**E**), and 1000 nm (**F**) PS particles (*n* = 5 mice per group), each scattered dot on the bar graph represents one individual mouse. Data are presented as the mean ± SEM. Statistical significance was assessed using two-tailed unpaired Student’s *t*-tests in (**B**,**D**,**F**) and a two-tailed unpaired Welch’s *t*-test in (**E**). ns, not significant; ** *p* < 0.01 and **** *p* < 0.0001.

Initial lacteals contain discontinuous button-like junctions between adjacent lymphatic endothelial cells, which create entry portals for fluid and macromolecules, whereas continuous zipper-like junctions form a tighter barrier that restricts paracellular uptake [20]. Y-27632 treatment increased the ratio of zipper-like junctions and decreased the ratio of button-like junctions in the villus lacteals (Figure 5A,B). Y-27632-induced lacteal junction zippering was accompanied by reduced mesenteric lymph node fluorescence from the 500 and 1000 nm particles (Figure 5C–F). These findings support a functional contribution of lacteal junction organization to the uptake and downstream lymphatic transport of the larger PS particles.

### 3.6. Repeated Oral Exposure Altered Body Weight Trajectories and Mesenteric Lymph Node Morphology

**Figure 6:**
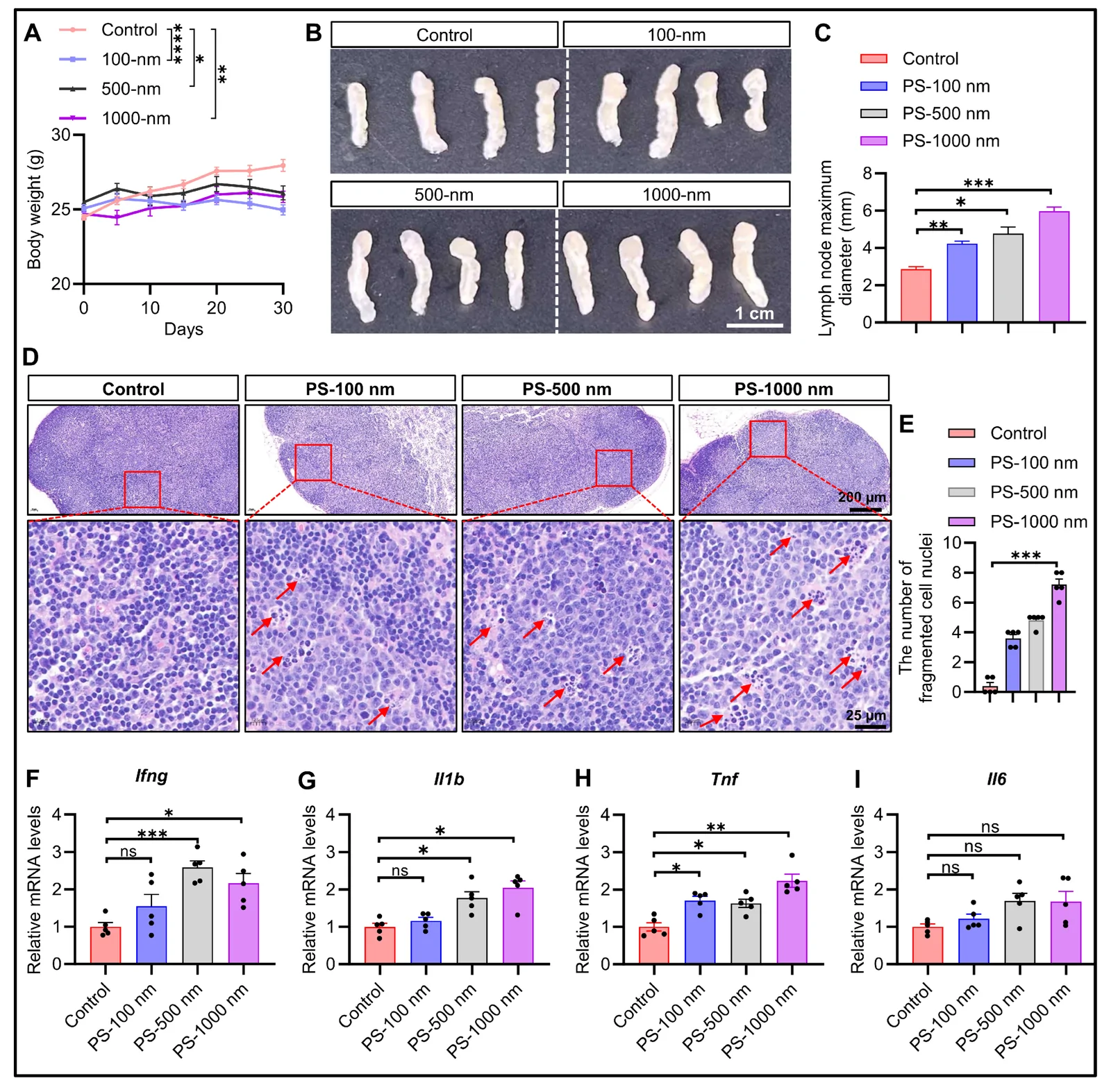
Repeated oral exposure to PS particles alters body weight and mesenteric lymph node morphology: (**A**) Body weight trajectories of mice during daily oral exposure to 15 mg/kg PS particles for 30 d (*n* = 10 mice per group). (**B**) Representative images of mesenteric lymph nodes collected after 30 d of oral exposure. (**C**) Quantification of the maximum lymph node diameter shown in (**B**) (*n* = 4 mice per group). (**D**) Representative hematoxylin and eosin (H&E)-stained sections of mesenteric lymph nodes collected after 30 d of oral exposure; Red arrows mark damaged cells with disorganized structure, focal vacuolation, widened intercellular spaces and fragmented nuclei. scale bar, 25 µm. (**E**) Quantification of cells with disrupted nuclei in (**D**) (*n* = 5 mice per group), each scattered dot on the bar corresponds to one individual mouse. (**F**–**I**) Relative mRNA expression of the pro-inflammatory cytokine genes *Ifng* (**F**), *Il1b* (**G**), *Tnf* (**H**) and *Il6* (**I**) in mesenteric lymph nodes after repeated exposure to PS particles of different sizes, as determined by qRT-PCR. Expression levels were normalized to those of the control group (*n* = 5 mice per group), each scattered dot on the bar graph represents one individual mouse. The data are presented as the mean ± SEM. In (**A**), body weight data were analyzed using repeated-measures two-way ANOVA followed by Sidak’s multiple-comparison test, with post hoc comparisons between each PS-particle group and the PBS-treated control group performed on day 30. Data in (**C**,**F**–**I**) were analyzed using Brown–Forsythe and Welch ANOVA followed by Dunnett’s T3 multiple-comparison test, whereas data in (**E**) were analyzed using the Kruskal–Wallis test followed by Dunn’s multiple-comparison test. ns, not significant, * *p* < 0.05, ** *p* < 0.01, *** *p* < 0.001 and **** *p* < 0.0001.

After 30 days of daily oral exposure, the body weight of mice in all PS-particle groups was lower than that of PBS-treated controls, with the greatest difference observed in the 100 nm group (Figure 6A). The maximum diameter of mesenteric lymph nodes increased most prominently in the 1000 nm group (Figure 6B,C). Histological examination revealed cellular disorganization, focal vacuolization and enlarged intercellular spaces in the exposed groups. Quantification further showed increased numbers of cells with disrupted nuclei, with more pronounced alterations in the 100 and 1000 nm groups (Figure 6D,E). Repeated PS-particle exposure was also associated with significant increases in the mesenteric lymph node mRNA expression of the pro-inflammatory cytokine genes *Ifng*, *Il1b* and *Tnf* (Figure 6F–H). *Il6* mRNA expression showed an upward trend, although the increase did not reach statistical significance (Figure 6I). Together, these morphological and transcriptional changes were consistent with a local inflammatory response following repeated PS-particle exposure.

## 4. Discussion

**Figure 7:**
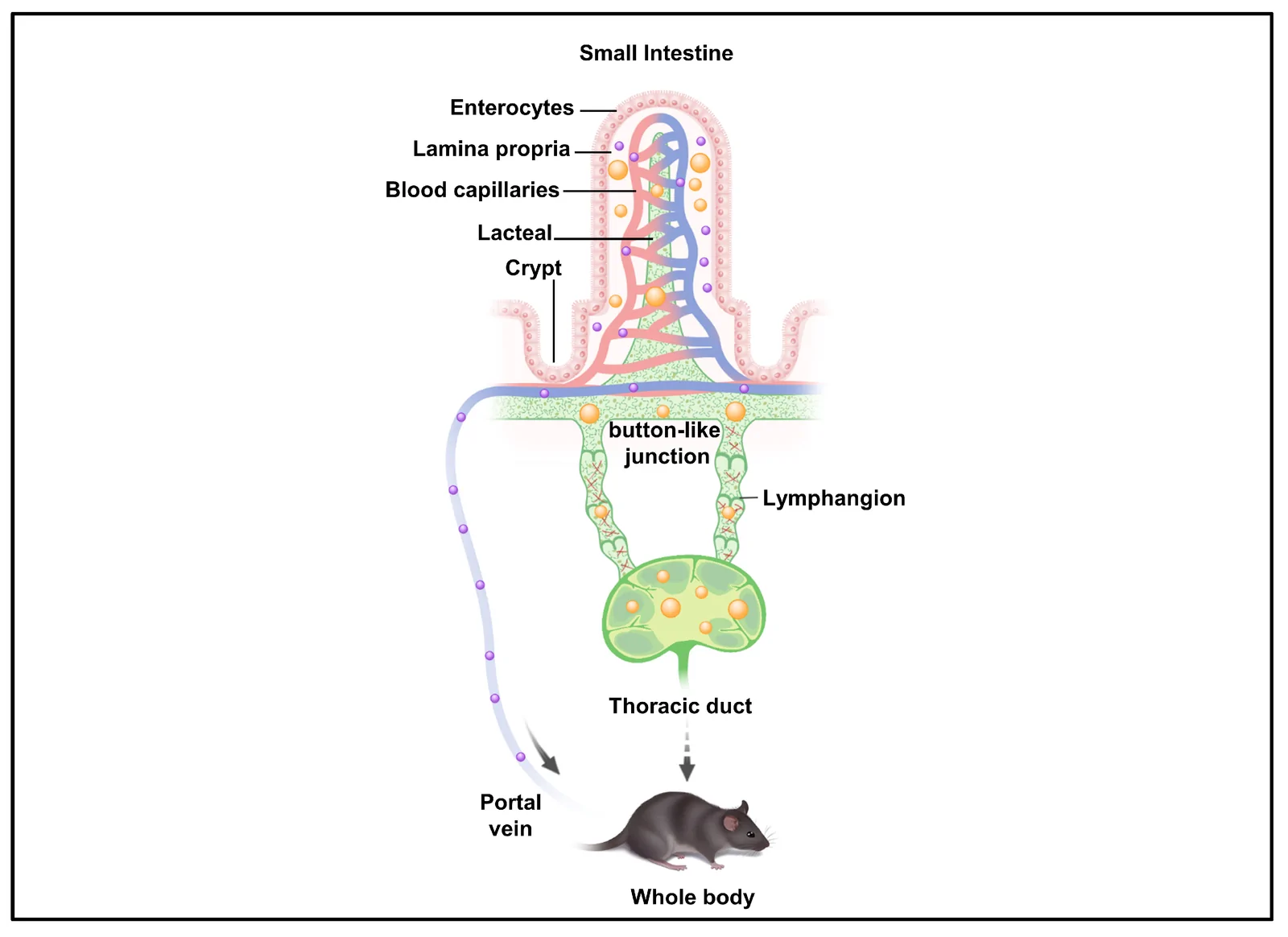
Proposed model of size-associated intestinal transport of PS particles. Schematic representation of the transport patterns observed for 100, 500 and 1000 nm PS particles after oral administration. The 100 nm particles preferentially localized to villous blood capillaries and appeared rapidly in the circulation and systemic organs. In contrast, the 500 and 1000 nm particles preferentially localized to lacteals and downstream mesenteric lymphatic structures, a pattern consistent with lymphatic transport contributing to their delayed systemic appearance.

Our study revealed size-associated partitioning of orally administered PS model particles between the vascular and lymphatic compartments of intestinal villi. Rather than producing a simple increase or decrease in overall uptake, the three particle formulations showed distinct spatial and temporal distribution patterns. The 100 nm particles localized predominantly to villous blood capillaries and appeared earlier in the blood, liver and kidneys, whereas the 500 and 1000 nm particles were more frequently associated with lacteals and downstream mesenteric lymphatic vessels and showed delayed systemic appearance. These findings support a model in which intestinal route selection contributes to the timing and anatomical pattern of systemic particle distribution (Figure 7).

Previous studies have mainly examined how plastic particles cross the intestinal epithelium. Caco-2-based monocultures and more complex mucus- and M-cell-containing models show that PS-particle association, internalization and transepithelial transport vary with particle size and cellular composition [14,45]. In vivo imaging has also detected 20 and 40 nm PS nanoparticles in the villus lamina propria and in vascular and lymphatic structures following epithelial uptake [46]. These studies demonstrate that ingested particles can reach both compartments but do not establish whether different sizes preferentially partition between adjacent villous blood capillaries and lacteals. Organ-level studies likewise cannot identify the intestinal route through which particles enter the circulation [47]. By tracking particle-associated fluorescence from jejunal villi through the mesentery to mesenteric lymph nodes, our study links local route preference with downstream distribution kinetics. The early blood and organ peaks of the 100 nm particles are consistent with relatively rapid vascular transport, whereas the delayed peaks and stronger lymph node signals of the larger particles are consistent with intestinal lymphatic transport [48]. These findings extend previous work on epithelial uptake by identifying a subsequent, size-associated transport step within the intestinal villus.

The Y-27632 experiment provides functional support for lacteal involvement in transporting the larger particles. Initial lymphatic capillaries contain discontinuous button-like junctions that permit fluid and macromolecular entry, whereas continuous zipper-like junctions form a more restrictive barrier [23,49]. In intestinal lacteals, pharmacological or genetic induction of junction zippering reduces chylomicron entry and mesenteric lymphatic transport [50,51]. Consistent with this mechanism, Y-27632 increased the proportion of zipper-like junctions and reduced the mesenteric lymph node accumulation of 500 and 1000 nm particles [20], whereas no significant reduction was observed for the 100 nm particles. This differential response supports a greater contribution of lacteal transport to the uptake and downstream distribution of the larger particles and extends the potential cargo regulated by lacteal junction organization from endogenous lipid-rich particles to exogenous submicrometer materials [52,53].

Although particle size provides a useful framework for interpreting these transport patterns, it was not the only physicochemical property that differed among the formulations. The negative surface charge may be associated with sulfate-containing end groups introduced during particle synthesis [54]. The increasing magnitude of the negative zeta potential with particle size may reflect size-associated electrokinetic effects, including electrical double-layer relaxation and hydrodynamic retardation, together with formulation-related differences in surface groups [55,56,57,58,59,60]. The increases in hydrodynamic diameter and PDI, together with the reduced magnitude of the negative zeta potential in 10% FBS, may reflect serum-protein adsorption and the potential formation of a biomolecular corona, which could alter the apparent size, surface properties and dispersion behavior of the particles [61]. Although the distributions broadened, all three formulations retained a single dominant peak without a distinct secondary population of large aggregates. Therefore, the observed vascular–lacteal partitioning should be interpreted as size-associated within the tested particle set, with differences in surface charge, aggregation state and acquired biomolecular coatings potentially contributing to the observed transport behavior.

Repeated exposure revealed distinct biological response profiles across the tested particle sizes. The greatest reduction in body weight gain occurred in the 100 nm group, whereas mesenteric lymph node enlargement was most prominent in the 1000 nm group. Histological alterations were also more pronounced at both ends of the tested size range. These nonmonotonic responses argue against a simple model in which smaller particles are uniformly more harmful [62]. Instead, these findings raise the possibility that the more rapid systemic distribution of smaller particles and the stronger lymphatic association of larger particles may produce different systemic and local exposure burdens during repeated exposure [63]. In line with this possibility, lymph node enlargement and histological alterations were accompanied by increased *Ifng*, *Il1b* and *Tnf* mRNA expression, a pattern consistent with a local inflammatory response. However, the present findings do not establish a direct causal relationship between the transport patterns observed after single-dose administration and the effects observed after repeated exposure.

The toxicological findings should also be interpreted in the context of environmental exposure and the experimental dose. PS micro- and nanoplastics have been reported in food, drinking water and other media relevant to human ingestion [64,65,66]. The 15 mg/kg dose used in the single-dose experiment and the 15 mg/kg/day dose used for repeated exposure were selected based on comparable oral PS-particle models [17,33,34,35]. For context, a previously estimated upper human micro- and nanoplastic intake of 5 g/week corresponds to approximately 11.5 mg/kg/day for a 62 kg adult [33,34,67], although substantially lower estimates have also been reported [68]. Because current estimates vary with the sampled environment, analytical method and exposure assumptions, the present dose represents a controlled high-exposure condition for evaluating particle transport and biological responses. Extrapolation to human health should account for differences between these experimental conditions and typical environmental exposure.

The fluorescence-based approach was selected to resolve the spatial relationships between PS particles, villous blood capillaries and lacteals. Its histological resolution and compatibility with vascular and lymphatic immunostaining enabled direct colocalization within individual villi. Positron emission tomography (PET) provides sensitive whole-body quantification but requires radiolabeling and lacks sufficient spatial resolution to distinguish adjacent capillaries and lacteals [69,70]. Magnetic resonance imaging (MRI) permits non-ionizing deep-tissue imaging, although magnetic modification may alter particle properties [71]. Raman and stimulated Raman scattering provide label-free, polymer-specific identification, whereas current photoacoustic imaging of microplastics relies on the optical absorption of plastic-associated pigments to enable longitudinal detection in living tissues [72,73,74,75]. These methods provide complementary information but are less readily integrated with immunofluorescence-based localization of villous structures. Fluorescence imaging was therefore appropriate for the present anatomical question. Its limitations include potential fluorophore leakage, tissue autofluorescence, optical attenuation, the lack of absolute particle quantification and the absence of independent polymer-specific confirmation. Because these approaches were not evaluated under matched conditions, this comparison is qualitative rather than a quantitative performance benchmark.

Several limitations define the scope of these findings. The commercially available fluorescent spherical PS particles provided a standardized system for comparing transport patterns but did not reproduce the irregular morphology, surface oxidation, additive composition and adsorbed contaminants of environmentally aged plastics [76]. Weathering and gastrointestinal digestion may alter particle surface chemistry, aggregation and biological interactions; environmentally derived particles may therefore behave differently [76]. Particle behavior was characterized in pure water and 10% FBS but not in simulated gastric or intestinal fluids. Moreover, although inflammatory cytokine-gene expression was examined, protein-level cytokines, immune-cell composition and lymph node immune function were not evaluated. Future studies combining environmentally aged particles, simulated gastrointestinal conditions, independently controlled surface properties, polymer-specific detection and immune profiling will help establish the environmental relevance and biological consequences of these transport patterns.

## 5. Conclusions

In summary, orally administered PS model particles exhibited size-associated partitioning between two intestinal transport routes. The 100 nm particles were preferentially associated with villous blood capillaries and appeared rapidly in the blood and systemic organs, whereas the 500 and 1000 nm particles were more strongly associated with lacteals and accumulated along the mesenteric lymphatic pathway. Modulation of lacteal junction organization reduced the mesenteric lymph node accumulation of the larger particles, supporting a functional contribution of lacteal permeability to their downstream transport. These findings identify the intestinal blood–lacteal interface as an important site influencing particle fate and provide a framework for investigating the size-associated systemic and lymphatic distribution of ingested particulate contaminants.

## Data Availability

The original contributions presented in this study are included in the article. Further inquiries can be directed to the corresponding author.

## Abbreviations

The following abbreviations are used in this manuscript:

PS	Polystyrene
MPs	Microplastics
NPs	Nanoplastics
PS-NPs	Polystyrene nanoparticles

## Appendix A

**Table A1:** Antibodies used for immunofluorescence staining.

Antibody	Species	Vendor	Cat. No.	Sample Fixation	Dilution
LYVE-1	Rabbit anti-mouse	AngioBio	11-034	Mouse tissue fixed in 4% formaldehyde for 6 h or 1.6% formaldehyde for 43 min	1:800
Endomucin	Rat anti-mouse	Hycult	HM1108	Mouse tissue fixed in 4% formaldehyde for 6 h	1:250
VE-cadherin	Rat anti-mouse	BD	555289	Mouse tissue fixed in 1.6% formaldehyde for 43 min	1:300
VEGFR3	Goat anti-mouse	R&D Systems	AF743	Mouse tissue fixed in 4% formaldehyde for 6 h	1:300
Alexa Fluor™ 488-conjugated	Donkey anti-rat IgG	Invitrogen	A21208	**_**	1:1000
Alexa Fluor™ 546-conjugated	Donkey anti-rabbit IgG	Invitrogen	A10040	**_**	1:1000
Alexa Fluor™ 647-conjugated	Donkey anti-rat IgG	Invitrogen	A78947	**_**	1:1000
Alexa Fluor™ 488-conjugated	Donkey anti-goat IgG	Invitrogen	A32814	**_**	1:1000

**Table A2:** Sequences of primers used in qRT-PCR analysis.

Gene Name	Primer Sequence (5′ to 3′)
Mus-*Actb*-F	TGTGACGTTGACATCCGTAAAG
Mus-*Actb*-R	GCAGTAATCTCCTTCTGCATCC
Mus-*Ifng*-F	CAGCAACAGCAAGGCGAAAAAGG
Mus-*Ifng*-R	TTTCCGCTTCCTGAGGCTGGAT
Mus-*Il1b*-F	ATGCCACCTTTTGACAGTGATG
Mus-*Il1b*-R	TGTGCTGCTGCGAGATTTGA
Mus-*Tnf*-F	ACGGCATGGATCTCAAAGAC
Mus-*Tnf*-R	AGATAGCAAATCGGCTGACG
Mus-*Il6*-F	AGTTGCCTTCTTGGGACTGA
Mus-*Il6*-R	TCCACGATTTCCCAGAGAAC
